# Opportunities and Challenges in Cross-Country Collaboration: Insights from the Beneluxa Initiative

**DOI:** 10.3390/jmahp12030012

**Published:** 2024-07-09

**Authors:** Zilke Claessens, Michiel Lammens, Liese Barbier, Isabelle Huys

**Affiliations:** Department of Pharmaceutical and Pharmacological Sciences, KU Leuven, 3000 Leuven, Belgium

**Keywords:** Beneluxa initiative, cross-country collaboration, health technology assessment, International Horizon Scanning, pricing and reimbursement, EU HTA Regulation, joint HTA, semi-structured interviews

## Abstract

National pricing and reimbursement agencies face growing challenges with complex health technologies, prompting European policy advancements. Beneluxa is a cross-country collaboration involving Belgium, the Netherlands, Luxemburg, Austria, and Ireland that aims to address sustainable access to medicines. In view of the soon-to-be-implemented EU HTA Regulation, insights and experiences from stakeholders with Beneluxa cross-country collaboration could provide possible transferable learnings. Therefore, this research aims to (i) identify the opportunities and challenges faced by Beneluxa, (ii) gather insights from stakeholders, namely (possible) applicants and policymakers, within and beyond Beneluxa on the initiative and broader cross-country collaboration principles, and (iii) transfer these insights into learnings and recommendations in anticipation of the full implementation of the new HTA Regulation. Fifteen semi-structured interviews were conducted with industry and European HTA/policy stakeholders. The principal challenges discussed by stakeholders encompass hesitancy from the industry toward Beneluxa assessments, which were attributed to procedural and timeline uncertainties, legislative framework ambiguity, and challenges in terms of industry’s internal organization. Another challenge highlighted is the resource-intensive nature of the procedure due to diverse approaches among member states. In addition, industry stakeholders mentioned limited communication and procedural complexity. Despite challenges, both stakeholder groups recognized important opportunities for cross-country collaboration. Transferable insights for future cross-country collaboration include transparent communication, clear legislative embedding, internal industry restructuring to facilitate joint HTAs, and member state support for conducting collaborative assessments. The study underscores diverging views among stakeholders on cross-country collaboration’s potential to support HTA and the market access of complex health technologies. While acknowledging benefits, there still are challenges, including industry hesitancy, emphasizing the need for transparent communication and clear guidance in the evolving EU HTA landscape.

## 1. Introduction

As the pharmaceutical market gradually evolves from the development of one-size-fits-all medicines to more personalized and complex treatments [1], challenges arise during the value assessment and reimbursement decision making of these products. The increasing technological character of complex health technologies and often limited efficacy and safety data due to a small study population or the inability to perform studies that analyze long-term effects [1] complicate the value assessment by health technology assessment (HTA) bodies and national pricing and reimbursement (P&R) agencies [2,3]. Moreover, the high cost of these medicines brings extra uncertainty to the assessment considering the financial constraints of countries in terms of reimbursement of said medicines [2,3].

During recent years, some countries decided to cooperate in so-called HTA cross-country (CC) collaborations to help tackle budgetary and value assessment difficulties and cope with the uncertainties and workload that comes with it [2,3]. In addition, EU-wide initiatives like the EUnetHTA21 consortium (which was operational until September 2023) and the recent EU HTA Regulation (2021/2282) aimed to address these challenges [4,5]. The new EU HTA Regulation supports cooperation between member states by establishing measures for a joint clinical assessment (JCA) of health technologies. Notably, the HTA Regulation introduces a JCA to streamline the assessment process. More concretely, this means that a JCA will be conducted for promising health technologies, of which the outcome is to be considered in various individual member states. The economical part of the HTA will remain a national responsibility. The EU’s HTA Regulation represents a significant step toward harmonizing assessments and fostering collaboration among European member states.

**Figure 1:**
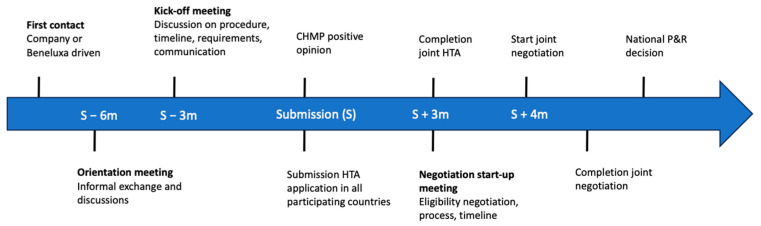
An overview of Beneluxa procedural steps and associated timeline. m = month, S = submission, CHMP = Committee for Human Medicinal Products, HTA = health technology assessment, P&R = pricing and reimbursement. Joint negotiations are subject to agreement of all parties and are not standard part of Beneluxa procedures.

On a regional level, Beneluxa is an example of an early and leading CC collaboration which started in 2015 with Belgium, the Netherlands, and Luxembourg and was soon complemented by Austria (in 2016) and Ireland (in 2018). The Beneluxa initiative aims at fostering patients’ access to innovative medicines at an affordable cost [6]. To achieve this aim, Beneluxa focuses on four areas of collaboration, namely on the level of (i) horizon scanning (identifying important pharmaceutical innovations before they reach the market, giving insights regarding expected costs and enabling timely decision making), (ii) information sharing and policy exchange, (iii) joint HTA including clinical and economic criteria, and (iv) joint P&R negotiations [6]. Beneluxa offers a voluntary alternative for the national market access pathway starting with a joint HTA where at least two member states participate. If agreed by all parties, joint HTA can be followed by joint negotiations where all parties together discuss the P&R of the health technology [6]. The legal decision on P&R, however, remains a national competence. Figure 1 shows an overview of Beneluxa’s procedural steps together with timelines.

Since the launch of the initiative in 2015, only a limited number of products, in total 14, have been assessed. For most of these products, Beneluxa countries collaborated on the level of information sharing and policy exchange. For four products, which all target rare diseases, joint negotiations took place. All the products that have been assessed via one of the collaboration areas of Beneluxa are listed in Supplementary Table S1. One of these products, a gene therapy medicine for treating spinal muscular atrophy and said to be one of the most expensive medicines worldwide [7], has successfully completed both joint HTA and joint negotiations, making it simultaneously available for patients in Belgium, the Netherlands, and Ireland [8]. In 2019, the European Federation of Pharmaceutical Industries and Associations (EFPIA) published a paper around the policy principles on CC collaborations. EFPIA argued that CC collaborations still must prove their added value toward enhanced access of pharmaceuticals [9].

The Beneluxa initiative has driven significant advancements in horizon scanning within healthcare, aiming to leverage pooled resources and expertise across member countries for improved efficiency and effectiveness [10]. This collaborative effort has led to the establishment of the International Horizon Scanning Initiative (IHSI) [11]. Variations in reimbursement decisions exist among other HTA agencies such as NICE (UK), GBA (Germany), and HAS (France) [12]. These differences are influenced by legal and institutional regulations as well as varying evaluation methods and criteria [12]. Nevertheless, differing HTA procedures are also reported among member states included in Beneluxa [13]. Despite these differences, Vreman et al. (2023) underscore the feasibility and benefits of CC collaborations in HTA under the Beneluxa framework; their findings highlight the potential of harmonizing HTA processes across borders as a crucial step toward unified implementation across Europe [13]. Enhancing HTA methodologies and fostering increased collaboration among agencies and political institutions could facilitate the alignment of HTA practices across the EU. Considering the new EU Regulation on HTA, CC collaborations have the opportunity to share the hurdles and learnings they encountered. As the Regulation also includes conducting joint HTA and horizon scanning, Beneluxa’s experiences and insights could help inform the further implementation of the Regulation. Therefore, this research aims to (i) identify the opportunities and challenges faced by Beneluxa in the four areas of collaboration, (ii) gather insights from stakeholders, namely (possible) applicants and policymakers, within and beyond Beneluxa on the initiative and broader CC collaboration principles, and (iii) transfer these insights into valuable learnings and recommendations in anticipation of the full implementation of the new HTA Regulation [4].

## 2. Materials and Methods

In this study, semi-structured interviews were conducted with experts from the pharmaceutical industry and policymakers and advisors, including HTA representatives, P&R agencies, patient organizations, and other policymakers. Purposive sampling targeted individuals with expert insight, spanning the Beneluxa member countries (Belgium, the Netherlands, Luxembourg, Austria, and Ireland) and pan-European entities [14].

Interviews, held between February and April 2022, were conducted in English or Dutch based on interviewee’s preference, lasting between 26 and 75 min (on average 48 min). The semi-structured approach followed a topic guide which was developed based on scientific and grey literature (including policy documents, legislations, and policy statements). The topic guide consisted of 3 main parts, covering (i) the areas of cooperation, (ii) the current participation of the stakeholders in the initiative, and (iii) the future perspectives. The topic guide was refined through two pilot interviews conducted in February 2022, one for each stakeholder group. The pilot interviews resulted in minor changes in the formulation and order of questions and were subsequently included in the analysis.

Interviews were conducted via Teams^®^ and digitally recorded by at least two researchers. One researcher functioned as an interviewer (ZC, ML), and the other as functioned observer (ZC, ML, LB). Recordings were transcribed verbatim and pseudonymized. Interviews were conducted until data saturation was reached, which was determined through the code frequency count method with a stopping criterion of three consecutive interviews (see Supplementary Figure S1) [15]. In other words, data saturation was reached when there were no new codes arising for three consecutive interviews in chronological order of conduct. The transcripts were subsequently analyzed qualitatively using the thematic framework method with NVivo^®^ software (release 1.7.1) [16]. The data were thoroughly familiarized by reading and re-reading the transcripts and listening back to audio-recorded interviews when necessary to clarify any unclear parts. Analytical notes, thoughts, or impressions, especially those regarding strong or contrasting views expressed by interviewees, were written in the margins of the documents. Next, a thematic framework, including themes for coding, was developed in collaboration with all authors. 

Two researchers (ZC, ML) performed parallel coding in a blinded way and resolved discrepancies through consensus discussions. Finally, the framework matrix was qualitatively analyzed by two researchers independently (ZC, ML). A meeting was then organized for the research team to discuss these qualitative interpretations. Ethical clearance for the study was obtained from Ethics Committee Research UZ/KU Leuven (S66252).

## 3. Results

**Figure 2:**
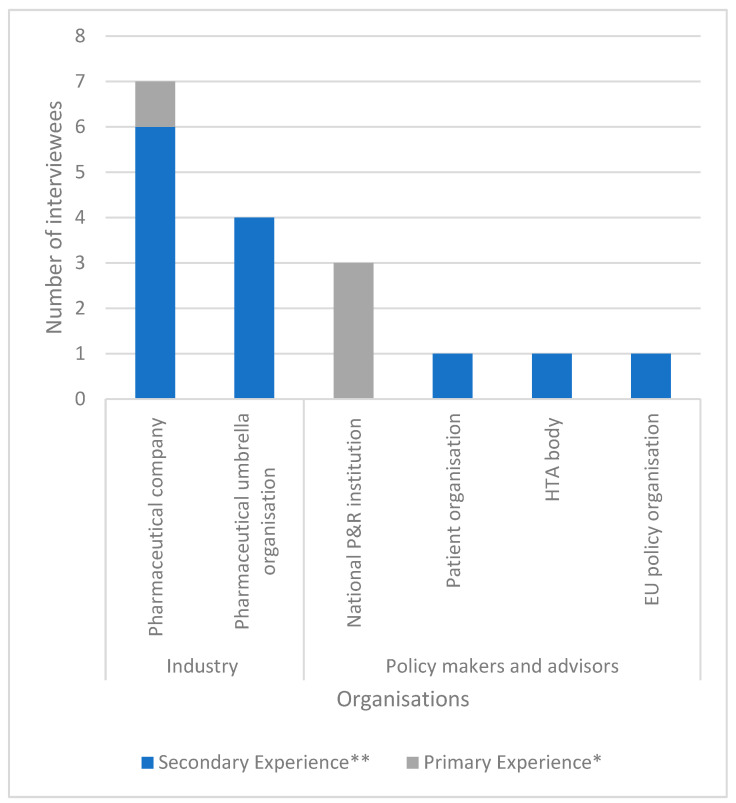
Interviewee characteristics. There were 15 interviews conducted with a total of 16 interviewees (interview IND5 consisted of two interviewees indicated with IND5a and IND5b). * Primary experience is active experience that the interviewees have obtained via applying for or reviewing a Beneluxa submission. ** Secondary experience is referring to experience that is gained via secondary sources such as scientific literature, conferences, and webinars.

A total of 34 stakeholders were invited to participate in this study. Fifteen semi-structured interviews with sixteen interviewees (Figure 2) were carried out to reach data saturation. Two participants were from Ireland, seven were from Belgium, two were from the Netherlands, and five were from regional (EU/Global) organizations. Stakeholder views on (1) the potential value of CC collaborations, (2) insights on hesitancy of industry to participate in the Beneluxa initiative, and (3) stakeholder’s suggestions for ways forward for future CC collaborations are described below. 

### 3.1. CC Collaboration in Theory: Stakeholders’ Perception of the Potential Value of CC Collaborations and the Case of Beneluxa

Both industry and policy interviewees perceived that CC collaborations, particularly the Beneluxa initiative, could significantly enhance the European market access framework. Interviewees recognized substantial advantages in terms of improving efficiency and quality compared to regular national assessments. These benefits highlighted by interviewees, primarily related to HTA and P&R assessments, are outlined below.

#### 3.1.1. Potential Efficiency Gains in HTA and P&R Assessments

Interviewees considered potential efficiency gains, for both applicants and assessors, to be the key advantage of CC collaborations. Conducting a joint CC assessment once for multiple member states, instead of individual assessments for each country, was highlighted as a time and resource-saving approach. Applicants would only need to prepare one dossier, and involved authorities could divide the work, hence reducing the workload for both parties. Within Beneluxa, the option for combined procedures of HTA and P&R negotiations was seen as a way to further enhance efficiency. Additionally, one policy interviewee explained that early dialogues with member states were considered beneficial, allowing applicants to adapt and harmonize evidence generation based on advice regarding different countries’ data and evidence requirements.

##### 3.1.2. Potential Quality Improvements in HTA and P&R Assessments

Beyond efficiency gains, interviewees suggested the potential for higher-quality assessments through CC collaborations. Unlike individual assessments conducted by each authority, CC assessments enable discussions on HTA and P&R views between national responsible authorities. Within Beneluxa, a case-by-case evaluation of procedural approaches is performed to finally identify the most suitable approach and optimize the value assessment of medicines. This collaborative approach, considering varied practices among participating countries, was seen as enriching evaluations through expertise-sharing and peer-review processes. Industry and policy interviewees supporting CC assessments argued that upon completion of the HTA, a more profound scientific evaluation would be available compared to a regular national assessment, further supporting P&R negotiations. This enhanced assessment of the product’s value could lead to well-informed medicine pricing particularly for complex and costly medicines. At the contrary, some industry interviewees perceived Beneluxa as a tool to lower medicine prices by countries increasing and leveraging negotiation power. 

### 3.2. CC Collaborations in Practice: Industry Hesitancy to Participate in the Beneluxa Initiative

While the theoretical advantages of the Beneluxa initiative in CC collaborations were acknowledged by industry and policy interviewees, industry interviews revealed hesitancy to engage in Beneluxa and mentioned that this was due to challenges in its practical implementation. As Beneluxa has limited pilot cases, industry experts expressed reservations in applying for a Beneluxa procedure. Challenges contributing to this hesitancy were outlined, including (i) uncertainty about procedures and timelines, (ii) an unclear legislative framework, and (iii) restrictions within pharmaceutical companies’ internal organizations.

#### 3.2.1. Uncertainty about Procedures and Timelines

Industry interviewees lacking applied experience with the Beneluxa procedure described it as a “black box” due to uncertainty about procedures and timelines. Assessor interviewees explained that Beneluxa allows higher procedural flexibility compared to a regular national procedure, since a case-by-case approach enables answering the needs of highly complex cases. Industry and assessor interviewees with hands-on experience with Beneluxa emphasized that during early communication between the applicant and participating authorities, procedures and predicted timelines are discussed and elucidated for the involved parties before initiation of the procedure. Some interviewees highlighted a perceived lack of transparency in communication, particularly by stakeholders without hands-on experience with the process. This lack of clarity raised concerns and hesitancy among industry interviewees. They emphasized the need for greater transparency in decision-making processes and timelines, which could be facilitated by the initiative itself or through shared experiences among applicants.

#### 3.2.2. Unclear Legislative Framework

A proposed solution by interviewees to address unclear procedures was embedding them in a legislative framework. Streamlining national requirements through legislation could alleviate administrative hurdles, enhancing efficiency. Interviewees suggested that legislative clarity should not only extend to procedures but also include implications for national P&R decisions. The absence of legislative embedding raised concerns among industry interviewees, who feared that decisions made within Beneluxa might not guarantee national implementation. 

#### 3.2.3. Restrictions in Terms of Pharmaceutical Company’s Internal Organization

Internal organization within pharmaceutical companies emerged as another factor influencing willingness to participate in Beneluxa. Large pharmaceutical companies typically organize market access activities at the national level, having trained teams to handle national P&R processes. The lack of a standardized procedure for CC submissions across national market access affiliates or at an overarching level complicates applying for a CC collaboration. Interviewees recommended central organization at the European headquarters level within pharmaceutical companies to decide upon and support CC applications, promoting a more streamlined approach.

### 3.3. Stakeholders’ Perspective on Future CC Collaboration

**Figure 3:**
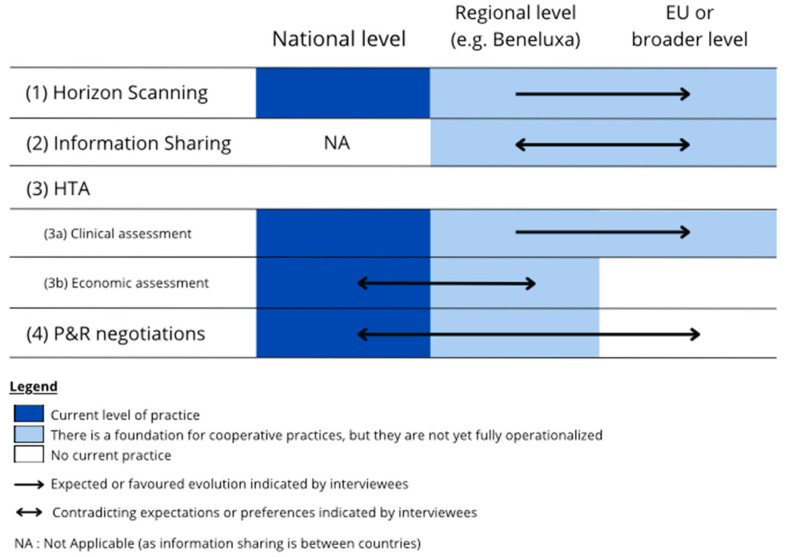
Summarizing figure on current practice and the interviewee-perceived application potential and preference of CC collaboration on three levels for four areas of cooperation. The color spectrum from blue to white indicates to what extent the area or cooperation is currently being executed on that level. Blue represents the level of cooperation that is currently common practice, lighter blue means that this level is not yet current standardized practice but fundaments are present, and white means that there is currently not yet any cooperation present on that level. The black arrow represents the possible evolutions that can be expected based on the interviews, providing that certain efforts or changes in the process are applied. As visually shown by the double arrows, the perspectives of interviewees differed or even contradicted for certain areas of cooperation, resulting in arguments for both a desired higher and lower level of cooperation. EU: European Union, HTA: health technology assessment, P&R: pricing and reimbursement.

Collaboration can be organized on three levels, being the national level, CC level (e.g., Beneluxa), or the broader pan-European or international level. In addition, within the Beneluxa initiative, there are four areas of collaboration: namely, (i) horizon scanning, (ii) information sharing and policy exchange, (iii) joint HTA including clinical and economic criteria, and (iv) joint P&R negotiations. Information sharing was identified as an overarching practice that is applied in all areas of collaboration with again area-specific challenges and opportunities. Figure 3 provides an overview of the current levels and areas of collaboration in market access procedures in Europe and interviewees’ preferences across these levels and areas of collaboration.

#### 3.3.1. Horizon Scanning

Horizon scanning, currently predominantly performed at the national level, is transitioning to more CC collaboration within initiatives like Beneluxa. Stakeholders perceived this shift positively, emphasizing the benefits of generating high-quality, detailed horizon scanning data to increase preparedness and enable early communication between regulatory and market access decision-makers. While acknowledging the value of early communication, concerns were raised, including the need for the careful interpretation and translation of data to national contexts. Industry interviewees suggested Beneluxa to take charge of this translation. Another concern pertained to the industry’s limited involvement in data collection and validation within the IHSI. Stakeholders proposed increased communication and collaboration to enhance the collection and validation of horizon scanning information. Policy interviewees noted IHSI’s intention to develop a database open for public consultation, addressing concerns about transparency and data validation.

#### 3.3.2. Information Sharing and Policy Exchange

Concerning the second pillar within Beneluxa, considerable ambiguity existed among interviewees with many stakeholders lacking awareness of the specific information encompassed by this pillar. In interviews, three potential types of information exchange emerged: 

(i) Exchange of expertise: One assessor interviewee described this as the sharing of knowledge, providing an avenue for member states within Beneluxa to exchange insights. Multiple interviewees highlighted how this information sharing enhanced their understanding of healthcare systems in different member states. The added value was highlighted by various stakeholders in the assessment of orphan medicines but also for CAR-T therapies and combination therapies. With regard to orphan medicines, one industry interviewee explained that often there are limited expert centers and patients per country, which makes it particularly interesting to share information between member states. Industry interviewees supported the exchange of knowledge as long as it would pertain to non-confidential information.

(ii) Exchange of policy practices: Policy exchange could be discussing the exchange of policy-related information, encompassing developments in regulations, reactions to evolving policies, and the collective shaping of the European policy agenda. 

(iii) Exchange of net prices: Industry interviewees predominantly referred to information sharing in the context of price negotiations. Sharing net prices among countries was perceived to have a substantial impact on the final product price in Europe through reference pricing. Some stakeholders, including assessors and those actively engaged in Beneluxa, welcomed the idea of increased transparency in pricing. However, it was clarified by two assessor stakeholders with active involvement in Beneluxa that the information shared did not include confidential prices; only publicly available information would be part of this exchange. 

#### 3.3.3. Joint Clinical Assessment 

Clinical HTA in Europe is predominantly performed at national level with CC collaborations like Beneluxa and EU collaborations such as EUnetHTA not yet standard practice. The shift toward more collaboration is perceived as a solution to address increasing technological complexity in medicines. However, interviewees with active Beneluxa experience reported higher workloads compared to national procedures due to alignment difficulties between healthcare systems. Challenges in streamlining methodologies and standards across member states were highlighted, particularly with the changing EU HTA framework. Furthermore, the EU HTA Regulation also raised questions about the place of CC clinical assessments, such as within Beneluxa, within the EU framework. Interviewees envisioned three possible scenarios for the future of CC joint clinical assessments within Beneluxa:

(i) In the first scenario, interviewees expected CC clinical assessments within Beneluxa to be replaced by collaborative practices at the EU-wide level, where CC collaborations, such as Beneluxa, will consider the EU JSCs (joint scientific consultations) and JCAs (joint clinical assessments) and take it further in national or CC economical HTA and P&R negotiations. These interviewees believe the future opportunity for Beneluxa lies in the joint economical HTA and price negotiations.

(ii) In the second scenario, interviewees identified a more complementary role where CC collaborations as within Beneluxa can function as active contributors to the EU JCA, which can at the same time assure translation to the CC level. 

(iii) In a third scenario, interviewees imagined for Beneluxa to be absorbed by the HTA Regulation, meaning that the joint clinical HTA is performed on the European level, and economic HTA and P&R negotiations stay at the national level. 

In all scenarios, learnings from existing CC collaborations, such as Beneluxa, could aid in developing collaborative HTA on the EU level as they gained experience on how to jointly conduct HTA.

#### 3.3.4. Joint Economic Assessment

While currently the economic part of the HTA is mainly conducted nationally, it is also an option within Beneluxa. Most industry and assessor interviewees explained their preference for keeping the economic HTA at the national level. This stems from the explanation that pharmaco-economic analysis is heavily determined by country-specific epidemiological and economic factors and hence difficult to generalize across countries. However, some expressed that if these differences could be minimized, collaboration on economic HTA could be favorable and lead to high-quality assessments. Similar to joint clinical HTA, joint work on economic HTA was believed by some to potentially lead to increased efficiencies in time and resources.

#### 3.3.5. Pricing and Reimbursement Negotiations

P&R negotiations predominantly occur at the national level, with joint P&R negotiations within Beneluxa being uncommon. Stakeholders presented diverse opinions on the preferred negotiation level, referencing the European-wide procurement of COVID-19 vaccines as an example of a more open environment for joint negotiations, which was deemed especially useful in emergencies. Some favored CC collaboration, believing it could contribute to a more objective value assessment and a fairer price. Joint negotiations were seen as a means to objectify assessments, addressing concerns about potential bias in national settings where assessors and applicants may have informal relationships. Beyond enhancing assessment quality, CC collaboration was viewed as a way to achieve more equitable access across different member states. Suggestions were made for a system to calculate fair prices per member state based on their varying gross domestic products.

However, arguments against CC collaboration in P&R negotiations included concerns about increased workload due to procedural alignment difficulties, confidentiality issues related to net price sharing, and perceived imbalances in negotiation power. Stakeholders recognized the absence of a one-size-fits-all answer for determining the appropriate level of P&R negotiations, emphasizing its dependence on the product and applicant profile. Joint assessments were deemed most favorable for expensive medicines with high uncertainty, such as orphan medicines, especially when such uncertainty is combined with elevated prices, and in situations of small patient populations that make evidence gathering challenging. Additionally, some stakeholders saw more advantages for small and medium-size enterprises in joint P&R negotiations, citing differences in organizational structures and strategic approaches compared to larger pharmaceutical companies.

## 4. Discussion

The evolving landscape of HTA in Europe, marked by the new HTA Regulation and the establishment of the IHSI, signifies an important shift toward a more collaborative environment at the EU level. Notably, the year 2025 brings a pivotal milestone with the introduction of JCA for oncological medicines as well as Advanced Therapy Medicinal Products (ATMPs), mirroring the type of medicines previously assessed within the Beneluxa CC collaboration. This study investigated the dynamics of CC Collaboration assessment initiatives, drawing from Beneluxa as a case study, providing insights into the challenges and opportunities of joint HTA. While stakeholders expressed their belief in the potential opportunities and benefits of CC collaborations to enhance the efficiency and quality of assessments, these seem to have not fully materialized yet in practice within Beneluxa. The study offers nuanced perspectives from both assessors and applicants. 

While interviewees in this study identified increased efficiency as a major theoretical benefit of CC collaboration, they argued that this seemed to have not yet materialized in practice. This is in line with O’Mahony et al., who argued earlier that CC collaboration resulting in a reduction in time of joint HTA is unlikely, considering the differences in methods applied in the different member states [17]. Furthermore, interviewees perceived the collaborative potential to be highest in terms of horizon scanning, information sharing and policy exchange, and clinical HTA but hesitancy exists among industry and HTA/P&R interviewees regarding the joint economic assessment and P&R negotiations, which is in line with the findings of the qualitative analysis of Kleijnen et al. [18]. 

The findings of this study offer several practical applications. Firstly, the identification of procedural uncertainties, legislative ambiguities, and resource-intensive processes within Beneluxa provides actionable insights for policymakers at the EU level and member state level who are involved in CC collaborations. By addressing these challenges, stakeholders can enhance the efficiency and effectiveness of collaborative assessments. Additionally, the emphasis on transparent communication and clear legislative embedding serves as a practical guide for other JCA and CC collaborations. These insights help in streamlining processes, reducing hesitancy from the industry, and fostering a more cooperative environment among member states. Moreover, the recommendations for internal industry restructuring and member state support are directly implementable strategies that can facilitate smoother joint HTAs and better resource allocation. More specific recommendations per area of collaboration are discussed in the following sections. 

### 4.1. Recommendations for Horizon Scanning

Most interviewees consider horizon scanning as an important tool to collect useful information to prepare national healthcare systems with the arrival of new technologies and possibly accelerate the time-to-market. However, according to a 2022 report, only six countries (Iceland, Italy, the Netherlands, Norway, Sweden, and the United Kingdom) in the WHO European region have implemented a systematic horizon scanning system, which is possibly due to its resource intensiveness [10]. CC collaboration can save resources and hence offer a solution to this problem, potentially resulting in more efficiently carried out and qualitative horizon scanning reports. However, industry interviewees highlighted that they would like to be more involved during and after the data collection to validate and avoid the misinterpretation of data. Methodologies to implement the outcome of horizon scanning within subsequent HTA assessments warrant further exploration [19]. 

### 4.2. Recommendations for Information Sharing 

The findings of this study underline that it is important that the goal and the content of the information that is being shared is clear for all parties not only for the applicants but also for the wider public to create trust. The systematic sharing of data could support decision making; therefore, the WHO in collaboration with various EU member states started in 2022 the Novel Medicines Platform (NMP) with the aim of increasing transparency and the systematic sharing of access-related information [20]. In view of clinical study information sharing, the new clinical trial Regulation introduced the CTIS, which is a system intended to streamline clinical trial application processes and to increase transparency [21].

### 4.3. Recommendations for the Joint Clinical Assessment

The lessons drawn from the Beneluxa initiative offer valuable insights into shaping recommendations for successful JCA implementation. A comparison between learnings from the Beneluxa initiative and the HTA Regulation (EU) 2021/2282 is shown in Table 1. 

Transparency is crucial, demanding clear communication from both the policy and applicant/industry side [21]. The existing guidelines established by EUnetHTA21 for JCA already offer clear reporting of timelines, showing a commitment to transparency [22]. Despite this, uncertainties persist regarding JCA’s impact on assessment timelines in member states, raising concerns about potential negative influences [23]. With regard to the procedures, the literature reports various uncertainties perceived by stakeholders relating to the choice of comparators, the selection of reliable and clinically relevant endpoints or outcomes, and the management of uncertainty [24,25,26]. To generate trust, clear and transparent communication is needed on these procedural aspects of JCA and how to address issues of different methodologies, tools, and models for HTA across Europe [27]. 

The new HTA Regulation mandates member states to consider JCA reports nationally, yet questions remain about harmonizing implementation [26]. The inefficient utilization of JCA reports at the national level could hinder patient access, necessitating proactive measures. For instance, the 2022 Roadmap for the revision of reimbursement processes in Belgium, developed through extensive multi-stakeholder consultation, offers a legal basis for Beneluxa framework integration, emphasizing the importance of harmonizing European legislations at the national level [28,29,30].

Pharmaceutical companies’ market access departments are often organized at the national level, which might complicate participation in CC initiatives such as Beneluxa and also readiness for the soon to be implemented HTA Regulation. Internal restructuring of processes might be needed, and trade organizations could have a supportive role in this.

Lastly, insights from the policymaker interviews in this study and literature underscore together the necessity for member states to secure additional resources (i.e., monetary and staffing), particularly until the JCA harmonizes with national procedures [31,32]. Other research reports that in the end, the staffing requirements for joint assessments are comparable to those for national assessments [18]. A recent multi-stakeholder study by Julian et al. emphasized that the true added value of collaborative efforts within the EU HTA Regulation depends significantly on the development of effective methodologies and processes to overcome the anticipated challenges [26]. 

### 4.4. Recommendations for Joint Economic Assessment and Price Negotiations

In contrast with the previous areas, views and the willingness to collaborate on joint economic assessment and negotiations were more contested and heterogeneous. While no concrete recommendations were derived in this study for joint economic assessment and price negotiations, some experts expressed that assessing clinical and economic aspects separately may be inefficient. The successful joint procurement on vaccines during the COVID-19 pandemic might have led to a more positive opinion on negotiating together [33]. An important challenge identified in our study is the industry’s perception that CC collaborations are primarily tools for lowering medicine prices. Additionally, there is significant hesitancy from the industry to engage in these collaborations due to concerns about the sharing of confidential information, particularly regarding net prices. The fear is that such disclosure could negatively impact their profit margins through the reference pricing system [33,34,35]. 

### 4.5. Strengths, Limitations, and Future Research

The study employed semi-structured interviews, providing nuanced insights into CC collaboration in HTA from diverse stakeholder perspectives, drawing from the concrete case of Beneluxa. By engaging both industry/applicants and policymakers, it adopts an inclusive approach. Multiple researchers participated in data collection and analysis, minimizing potential biases. The alignment of the study with the introduction of the HTA Regulation enhances its timeliness, offering valuable insights for ongoing implementation. 

It should be noted that no Beneluxa representatives participated in this study, which poses an important limitation. Furthermore, some of the pharmaceutical industry interviewees did not have hands-on experience with Beneluxa. Since the study focused on experts from large pharmaceutical companies, it would be useful to explore in the future views of also small and medium-sized enterprises on CC collaboration. Notably, the absence of representatives from all Beneluxa member states poses a limitation. However, the inclusion of EU-level participants partially compensates, providing pan-European insights. The sample predominantly represents member states actively involved in Beneluxa, potentially limiting the generalizability of findings. Lastly, interviews were conducted at the beginning of 2022, and the healthcare sector can undergo rapid and unpredictable changes. Therefore at the time of publication, developments may have altered the HTA landscape; hence, future research should re-evaluate these insights considering ongoing and new developments.

Building on the insights gained from this research, investigating the harmonization between CC collaborations such as Beneluxa and the JCA at the EU level presents an important research area. Understanding how these two assessment processes can be complementary is essential for optimizing the evaluation and access pathways for innovative health technologies in Europe. By exploring the potential synergies, overlaps, and divergences between regional and EU-level assessments, researchers can provide valuable recommendations for streamlining and enhancing the overall HTA landscape.

## 5. Conclusions

This study investigated views of policymakers and the pharmaceutical industry about the Beneluxa CC collaboration. While participants recognized several important theoretical advantages that CC collaborations could bring in this area, these seemed to have not yet fully materialized in practice due to hesitancy from applicants, legislative and procedural uncertainty, and hurdles at the level of company’s internal organizational structure. Learnings from the Beneluxa initiative emphasize the importance of transparent and clear communication and guidance for successful CC collaboration in the field of HTA. Study findings might be relevant and informative for other CC EU activities and the evolving EU HTA landscape. 

## Figures and Tables

**Table 1:** Overview of the key qualitative stakeholder learnings from the Beneluxa initiative, evaluation of its reflection in the HTA Regulation and additional recommendations, as emerged from the industry and policymaker interviews of this study.

Beneluxa Learning	HTA Regulation (EU) 2021/2282		Recommendations
	Benefit	Challenge	
Need for clear insights in timelines and procedures to reduce applicant hesitancy	Expected timelines are provided Pilot studies can bring clarity	Unclear translation to timelines at the national level Uncertainty about exact procedures	Generate clarity on the translation to national timelines Transparent communication on the assessment procedures
The perceived ambiguity surrounding the legislative framework causes applicant hesitancy	Legal foundation Obligation for member states to consider the JCA	Uncertainty on harmonization of implementation of JCA report across member states Inefficient use of the JCA report at the national level could further delay market access	Create transparency on the impact of the JCA on national procedures Ensure clarity on the integration of the JCA within national frameworks
Companies’ internal organization is predominantly nationally organized		No guidance available for companies	Create guidance by sector organizations for companies to reorganize their internal structures Facilitate experience sharing between companies
Inefficient procedures, more resource intensive compared to the national assessment	Proposed implementing acts: national HTA representatives jointly discuss and exchange information and experiences		Provide additional support to allow smooth harmonization with national procedures

## Data Availability

The datasets presented in this article are not readily available because privacy restrictions of participants.

## Supplementary Materials

The following supporting information can be downloaded at https://www.mdpi.com/article/10.3390/jmahp12030012/s1, Figure S1: List of products (n = 14) assessed within Beneluxa to date (02/2024); Table S1: Timing of code development for interviews combined with respective saturation graph to illustrate data saturation.
