# Supplementary material for: Opportunities and Challenges in Cross-Country Collaboration: Insights from the Beneluxa Initiative
Source: J Mark Access Health Policy. 2024 Jul 9;12(3):144–57. doi: 10.3390/jmahp12030012 (PMC11270242; doi:10.3390/jmahp12030012)
Supplement: Supplementary file 1 [file jmahp-12-00012-s001.zip › jmahp-2979448-supplementary.pdf]

Supplementary Material

Table S1. List of products (n=14) assessed within Beneluxa to date (02/2024). Bold lines represent products that were subject of joint P&R negotiations and hence had the most extensive collaborative nature. API*: active pharmaceutical ingredient, EMA: European Medicines Agency, HTA: Health Technology Assessment, P&R: pricing and reimbursement, NL: the Netherlands, BE: Belgium, IR: Ireland, AU: Austria, LU: Luxemburg*

API	Year	Area of collaboration	Joint work
Lopitapide	2015	Joint HTA	Re-use NL HTA report by BE
Alirocumab	2015	Joint HTA	NL reviewer of BE HTA report
**Lumacaftor / ivacaftor**	**2016 & 2017**	**Joint HTA,<br>Joint P&R negotiations**	**Joint writing HTA report by NL & BE (NL reviewer)<br>Re-use HTA report by LU<br>Joint P&R negotiations**
Tafamidis	2017	Joint HTA	NL reviewer of BE HTA report<br>Re-use BE HTA report by LU
**Obeticholic acid**	**2017**	**Joint HTA**	**Joint writing HTA report by NL & BE**
Telotristat	2018	Joint HTA	Re-use NL HTA report by BE
Phenylbutyrate	2018	Joint HTA	Re-use NL HTA report by BE
Osimertinib	2018	Joint HTA	Re-use AU HTA report by BE
Abemaciclib	2018	Joint HTA	Re-use AU HTA report by BE
**Nusinersen**	**2018**	**Joint HTA, Joint P&R negotiations**	**Re-use NL HTA report by BE<br>Joint negotiations (NL & BE)**
Metreleptin	2019	Joint HTA	Joint writing HTA report by NL & BE (AU & IR reviewers)
Betibeglogene autotemcel	2021	Joint HTA	Joint writing HTA report by NL & BE (IR reviewer)
**Onasemnogene abeparvovec**	**2021**	**Joint HTA, Joint P&R negotiations**	**Joint writing HTA report by NL, BE & IR (BE & AU reviewers)<br>Joint negotiations**
**Atidarsagene autotemcel**	**2023**	**Joint HTA, Joint P&R negotiations**	**Joint writing HTA (BE, IR) + NL (co-author) & AU (reviewer)<br>Joint negotiations**

Supplementary Material

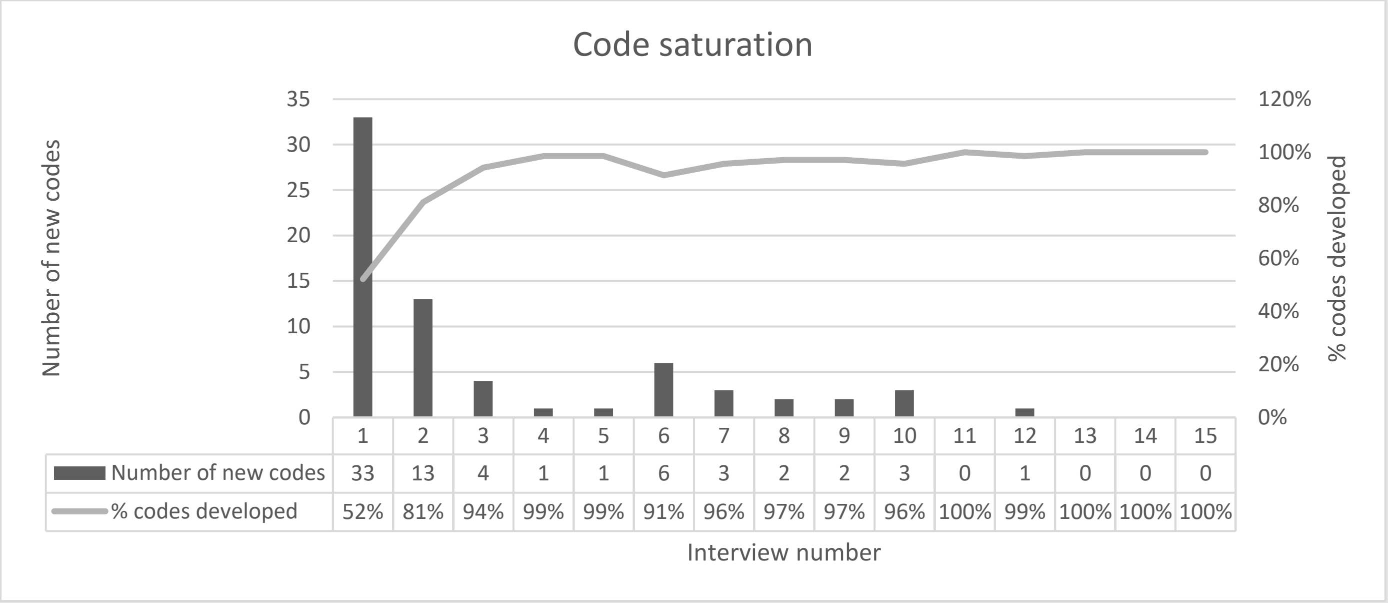

Figure S1. Timing of code development for interviews combined with respective saturation graph to illustrate data saturation
